# High rate of unexpected positive cultures in presumed aseptic revision of stiff shoulders after proximal humerus osteosynthesis

**DOI:** 10.1186/s12891-020-03430-y

**Published:** 2020-06-22

**Authors:** Doruk Akgün, Paulina-Maria Peters, Nina Maziak, Fabian Plachel, Marvin Minkus, Philipp Moroder

**Affiliations:** Center for Musculoskeletal Surgery, Charité - University Medicine Berlin, Freie Universität Berlin, Humboldt-Universität zu Berlin, and Berlin Institute of Health, Charitéplatz 1, D-10117 Berlin, Germany

**Keywords:** Positive culture, Proximal humerus fracture, Stiffness, Revision surgery

## Abstract

**Background:**

The aim of this study was to investigate the prevalence of positive microbiology samples after osteosynthesis of proximal humerus fractures at the time of revision surgery and evaluate clinical characteristics of patients with positive culture results.

**Methods:**

All patients, who underwent revision surgery after locked platting, medullary nailing or screw osteosynthesis of proximal humeral fractures between April 2013 and July 2018 were retrospectively evaluated. Patients with acute postoperative infections, those with apparent clinical signs of infection and those with ≤1 tissue or only sonication sample obtained at the time of implant removal were excluded. Positive culture results of revision surgery and its correlation with postoperative shoulder stiffness was analyzed in patients with an interval of ≥6 months between the index osteosynthesis and revision surgery.

**Results:**

Intraoperatively obtained cultures were positive in 31 patients (50%). Cutibacterium acnes was the most commonly isolated microorganism, observed in 21 patients (67.7%), followed by coagulase negative staphylococci in 12 patients (38.7%). There were significantly more stiff patients in the culture positive group compared to the culture-negative group (19/21, 91% vs. 15/26, 58%, *p* = 0.02). Furthermore, 11 of 12 (91.7%) patients with growth of the same microorganism in at least two samples had a stiff shoulder compared to 23 of 35 (65.7%) patients with only one positive culture or negative culture results (*p* = 0.14).

**Conclusion:**

Infection must always be considered as a possibility in the setting of revision surgery after proximal humerus osteosynthesis, especially in patients with postoperative stiffness.

## Background

Proximal humerus fractures are the third most common type of fracture [1]. Its incidence increases with an aging society and is estimated to triple by the year 2030 [2, 3]. Despite high frequency of this fracture, treatment remains a challenge and there is still no valid scientific evidence for the best treatment method [4]. A recent study showed the existence of a current tendency in stabilization of proximal humerus fractures with fixed-angle implants [5]. However, a notable number of postoperative complications with rates up to 36% leading to revision surgery in up to 25% of cases, are reported [6, 7]. Even during revision procedures for aseptic reasons, there is always suspicion that failure may have been the result of an undetected subclinical infection. Current literature in the field of periprosthetic joint infection suggests obtaining intraoperative cultures if infection is suspected to rule it out in all revision cases [8]. However there is little evidence available in literature on whether or not to obtain cultures in revision surgery after failed osteosynthesis.

Cutibacterium acnes has been implicated as a common microorganism associated with failures after shoulder surgery [9]. Obvious clinical signs of infection such as redness, swelling, sinus tract formation and fever are rarely encountered due to stealth type of clinical appearance of these low-grade infections [10]. The association between low-grade infection caused mostly by Cutibacterium acnes and a painful stiff shoulder is increasingly identified not only in patients undergoing shoulder arthroplasty but also shoulder arthroscopy [9, 11, 12]. Only few articles have been published dealing with infection after proximal humerus osteosynthesis and all of them are reporting only about acute postoperative infections [13, 14]. To our knowledge there is no data regarding the microbiologic profile of possible infections at the time of revision surgery without robust signs of infection after osteosynthesis of proximal humerus. Similar to the association with shoulder arthroplasty failure, low-grade infections may be associated with unexplained pain and stiffness after osteosynthesis of proximal humeral fracture.

The aim of this study was to investigate the prevalence of positive microbiology after osteosynthesis of proximal humerus fractures at the time of revision surgery and evaluate clinical characteristics of patients with positive culture results.

## Methods

### Patient selection and data collection

All patients, who underwent revision surgery after locked platting, medullary nailing or screw osteosynthesis of proximal humeral fractures between April 2013 and July 2018 were retrospectively evaluated. The study protocol was reviewed and approved by the institutional ethics committee.

A total of 135 consecutive patients were identified from the institutional shoulder database and included in the study. Patients with acute postoperative infections [15], those with apparent clinical signs of infection and those with ≤1 tissue or only sonication sample obtained at the time of implant removal were excluded.

The primary indication for revision surgery (e.g., stiffness, avascular necrosis, non-union, prominent hardware) was identified for each patient. Patient specific information such as age and gender at the time of revision surgery, the time interval between the osteosynthesis and revision surgery, clinical manifestation, preoperative range of motion, American Society of Anesthesiologist (ASA) score, surgical history of the involved joint, type of the osteosynthesis, laboratory values including C-reactive Protein (CRP) and serum leucocyte count as well as microbiological and sonication culture results of the revision surgery were recorded for all patients. Postoperative shoulder stiffness was defined as abduction < 90° and/or range of external and internal rotation < 60° in 0° abduction despite intensive physical therapy over a period of 6 months, as having stiffness in the first 6 months after surgery can be a normal part of the postoperative recovery process, while stiffness beyond 6 months is more likely to be permanent [6].

Furthermore, all radiographs of the affected shoulder obtained prior revision surgery were analyzed by the authors for non-union, malunion, and avascular necrosis as possible mechanical causes of a stiff shoulder [16].

### Microbiologic work-up

Reasons for obtaining cultures included suspicious history with unexplained pain and stiffness despite negative pre-operative work-up, intraoperative suspicion of possible infection by the surgeon and in some cases as a matter of routine. Tissue cultures were collected by a no-touch technique, were sent to the microbiology laboratory within 1 h and processed immediately. They were plated onto aerobic and anaerobic sheep blood agar plates and incubated for 14 days. Sonication was performed for 1 min at 40 kHz using a BactoSonic 14.2 unit (Bandelin, Berlin, Germany) as previously described [17]. The resulting sonication fluid was plated onto aerobic and anaerobic sheep blood agar plates and incubated also for 14 days. Attention was paid that patients had not received antibiotics within 2 weeks before revision surgery. Perioperative antibiotics were not given until all samples were obtained for culture analysis.

### Statistical analysis

Chi-squared and Fisher’s exact tests were used to find significant differences between categorical variables. The Mann Whitney U test (non-parametric) or two-sample *t*-test (parametric) was used to compare continuous variables between groups. The statistical subgroup analysis of stiffness was performed only with the patients with an interval of ≥6 months between the index osteosynthesis and revision surgery. The results were given as the mean and the standard deviation (SD) or as the number and percentage. A *p*-value < 0.05 was considered significant. SPSS version 20 (SPSS Inc., Chicago, Illinois) was used for the statistical analyses.

## Results

After excluding 12 patients due to acute postoperative infection and 61 patients due to insufficient microbiological data (≤ 1 tissue or only sonication sample obtained at the time of implant removal), 62 patients were subject of this study. The type of the osteosynthesis was proximal humeral locking plate in 54, intramedullary nail in six and screws in two patients.

The mean age of the study cohort at the time of revision surgery was 61.3 years (SD, 14.5 years, range 30–90 years) and 37 patients were females (59.7%). The mean interval and SD between the osteosynthesis of the proximal humeral fracture and revision surgery was 16.2 ± 20.5 months (Range 2–133) and 47 patients had an interval ≥ 6 months between the index osteosynthesis and revision surgery.

The most common clinical reason leading to revision surgery was shoulder stiffness in 34 patients. In 24 of these stiff patients radiographic evaluation revealed malunion (*n* = 4, 17%), nonunion (*n* = 2, 8%), avascular necrosis (*n* = 12, 50%) or a combination of these factors (*n* = 6, 25%) as a possible mechanical cause of stiffness. In the remaining 10 patients no mechanical cause for stiffness was identifiable.

Totally 195 tissue cultures were collected in 61 patients (3.1 tissue culture per case, SD, 1.3, range 2–8) and in 45 patients (73.8%) sonication of the removed hardware was performed. Intraoperatively obtained cultures were positive in 31 patients (50%). Cutibacterium acnes was the most commonly isolated microorganism, observed in 21 patients (67.7%), followed by coagulase negative staphylococci in 12 patients (38.7%). Polymicrobial results were present in 6 patients; all with isolation of Cutibacterium acnes. The detailed microbiological results of revision surgeries are summarized in Table 1. Thirteen patients with positive revision culture results had a growth of the same microorganism in at least two samples. Seven patients had two positive cultures, six patients with C. acnes and one patient with *Staphylococcus epidermidis*. Two patients had three positive cultures, one with C. acnes and one with Staphylococcus capitis. One patient had 4 positive cultures with *Staphylococcus aureus* and three patients had 5 positive cultures, all with C. acnes. Furthermore, the mean time to revision from the index surgery was significantly shorter in culture-positive group compared to culture-negative group (12.4 vs 20.5 months, respectively, *p* = 0.02).

**Table 1:** Microbiology of culture-positive group

Microorganisms	Number of patients, *n* = 31 (% of total patients
Cutibacterium acnes	21 (68)
CNS	
Staph. epidermidis	8 (26)
Staph. capitis	3 (10)
Staph. warneri	1 (3)
*Pseudomonas aeruginosa*	1 (3)
Staph. aureus	1 (3)
Paenibacillus pabuli	1 (3)
Corynebacterium spp.	1 (3)
Polymicrobial	6 (19)

*CNS* Coagulase-negative staphylococci

*Staph* Staphylococcus

Interestingly, there were significantly more stiff patients in the culture positive group compared to the culture-negative group, when including only patients with an interval ≥ 6 months between the index osteosynthesis and revision surgery (19/21, 91% vs. 15/26, 58%, *p* = 0.02). Furthermore, 11 of 12 (91.7%) patients with growth of the same microorganism in at least two samples had a stiff shoulder compared to 23 of 35 (65.7%) patients with only one positive culture or negative culture results. This difference was however statistically not significant (*p* = 0.14). Radiological evaluation of possible mechanical causes for stiffness did not show any significant differences in culture-positive and culture-negative groups and six stiff patients with positive cultures had unremarkable radiographs. Further demographic data and clinical and laboratory findings of the study cohort are summarized in Table 2. A further table summarizes the differences between patients with growth of the same microorganism in at least two samples and patients with only one positive culture or negative culture results (Table 3).

**Table 2:** The demographics and clinical characteristics for the culture-negative and culture-positive group

Variable	All patients, *n* = 62	Culture-negative group, *n* = 31	Culture-positive group, *n* = 31	*P*-value
Age at revision (yrs)^a^	61.3 ± 14.5	62 ± 14.6	60.6 ± 14.7	0.7
Gender^b^				0.12
Male	25 (40)	9 (29)	16 (52)	
Female	37 (60)	22 (71)	15 (48)	
Radiographic evaluation^b^				
Malunion	12 (19)	6 (19)	6 (19)	
Nonunion	5 (8)	3 (10)	2 (6)	
AVN	18 (29)	11 (36)	7 (23)	
Combination	13 (21)	4 (13)	9 (29)	
Negative	14 (23)	7 (23)	7 (23)	
Shoulder stiffness^b, c^	34/47 (72)	15/26 (58)	19/21 (91)	0.02
Serum CRP level (mg/l)^a^	5.4 ± 7.3	3.8 ± 3	6.8 ± 9.7	0.69
Time from index surgery until revision (months)^a^	16.2 ± 20.6	20.5 ± 24.4	12.4 ± 15.8	0.02
ASA score (median, range)	2 (1–3)	2 (1–3)	2 (1–3)	

*AVN* Avascular necrosis

^a^The values are given as the mean and the standard deviation. ^b^The values are given as the number with the percentage of the group in parentheses

^c^Including only patients with an interval of ≥6 months between the index osteosynthesis and revision surgery

**Table 3:** The demographics and clinical characteristics for the group 1 (patients with growth of the same microorganism in at least two samples) and group 2 (patients with only one positive culture or negative culture results)

Variable	Group 1, *n* = 13	Group 2, *n* = 49	*P*-value
Age at revision (yrs)^a^	55.8 ± 12.8	62.7 ± 14.7	0.12
Shoulder stiffness^b, c^	11/12 (92)	23/35 (66)	0.14
Serum CRP level (mg/l)^a^	5.2 ± 5.3	5.4 ± 8	0.95
Time from index surgery until revision (months)^a^	20.1 ± 22.9	15.2 ± 20.2	0.5

^a^The values are given as the mean and the standard deviation. ^b^The values are given as the number with the percentage of the group in parentheses

^a^Including only patients with an interval of ≥6 months between the index osteosynthesis and revision surgery

## Discussion

The evaluation for an implant-associated infection is a critical step of the preoperative work-up prior any revision surgery due to failed osteosynthesis. Although obvious infections after osteosynthesis with typical clinical signs, such as sinus tract, swelling, redness are easily detectable, occult infections can be a diagnostic challenge. Today, we have enough evidence that multiple cultures taken at the time of revision surgery for failed shoulder arthroplasty may be the most useful clinical tool in the diagnosis of periprosthetic shoulder infection [18]. Although a substantial number of studies were able to show a high prevalence of low-virulent microorganisms, especially Cutibacterium acnes, in shoulder arthroplasty and arthroscopy revision cases [9, 11, 19–21], our cohort study is the first study reporting the microbiological profile in the setting of revision surgery after osteosynthesis of proximal humerus fracture. Furthermore, we identified an association of positive culture results and shoulder stiffness after osteosynthesis of proximal humerus fracture.

Our study showed in a significant subset of patients undergoing revision surgery for indications other than acute infection a positive culture result, in almost two thirds of the patients with isolation of Cutibacterium acnes. Although Cutibacterium acnes might be considered as culture contaminant [22] or natural commensal of the shoulder [23], recent evidence showed a significant association between positive Cutibacterium cultures and failure after shoulder arthroplasty and arthroscopy [9, 11, 19, 21]. In these studies, pain and stiffness were the most common presentations in all patients with suspected low-grade infection. Similar to these findings Cutibacterium was the most isolated microorganism in our study cohort. Although the evaluated radiological signs such as avascular necrosis with a possible secondary screw penetration, non-union, or malunion can be attributed to shoulder stiffness, these factors were equally distributed in culture-negative and culture positive groups. Furthermore, in six stiff patients without any notable radiological signs cultures were positive. The well described ability of Cutibacterium acnes to manipulate the cellular response and therefore its role in chronic inflammation may explain the mechanical symptoms giving a classic postoperative frozen shoulder impression [11, 24, 25].

The precise evaluation for possible contamination of an osteosynthesis is even more important in the case of required revision arthroplasty. In a recent study Klatte et al. analyzed the incidence of positive pre-op aspiration and inflammatory markers in cases with shoulder arthroplasty for failed osteosynthesis [26]. In 4 of 17 preoperative aspirations bacterial growth was detected and the authors concluded that the risk of low-grade infection after osteosynthesis may be high and an adequate testing is recommended to rule out occult shoulder infection. However, it is not only important to rule out infection in cases with one stage revision from osteosynthesis to arthroplasty, but also in patients undergoing a subsequent shoulder arthroplasty at a second stage after months or even years after implant removal. Misdiagnosed occult infections in these cases can theoretically lead to prosthesis failure even after longer time periods.

This study has several limitations. The major drawback is the concern whether the detected microorganisms are indicative of an infection or possibly due to intra-operative skin contamination, although every possible precaution to avoid contamination was undertaken. Furthermore, because we do not have follow-up data, the relative pathogenicity of the different microorganisms could not be assessed. Despite the largest series of positive cultures reported in literature since now, our cohort is still small and can be underpowered. Further limitations are negative selection bias and the retrospective study design, which limits the reliability and completeness of our results. As a result, only almost in the half of all patients a microbiological work-up was performed and the study cohort represents only a subgroup of all patients undergoing revision surgery. The majority of the patients included in this study underwent revision surgery due to a complication after osteosynthesis and most of them were stiff. This fact did not allow us to do a multiple logistic regression model to determine the independent predictors of a stiff shoulder so the higher number of stiff shoulders in the positive culture group could be just a coincidence. However, this result is in concordance with the literature that Cutibacterium acnes is commonly isolated from cultures obtained from painful and stiff shoulders and may contribute to this clinical presentation. Although mostly microbiological growth of the same microorganism in at least two samples is considered as relevant for antimicrobial treatment [27], there is no consistent definition that determines whether a positive culture represent a “true infection” or a “contaminant” and the treatment for unexpected positive cultures in shoulder revision surgery remains unknown. Despite the fact that in our study patients with only one positive culture had also a stiff shoulder may only be a coincidence, it should be noted that small number of cultures might simply represent a lower quantity of bacteria present, which can indeed be relevant [28].

## Conclusions

A great subset of patients may have positive culture results in the setting of revision surgery after proximal humerus osteosynthesis, especially in patients with postoperative stiffness. Although the association between positive cultures and true infection remains unknown, infection must always be considered as a possibility in the setting of revision surgery despite radiological evidence of a possible mechanical reason.

## Data Availability

The datasets used and analysed during the current study are available from the corresponding author on reasonable request.

## Abbreviations

ASA: American Society of Anesthesiologist
CRP: C-reactive Protein
SD: Standard deviation
